# Molecular characterization of the rare translocation t(3;10)(q26;q21) in an acute myeloid leukemia patient

**DOI:** 10.1186/1755-8166-7-47

**Published:** 2014-07-15

**Authors:** Tereza Jancuskova, Radek Plachy, Lucie Zemankova, David Warren Hardekopf, Jiri Stika, Lenka Zejskova, Inka Praulich, Karl-Anton Kreuzer, Achim Rothe, Moneeb AK Othman, Nadezda Kosyakova, Sona Pekova

**Affiliations:** 1 Laboratory for Molecular Diagnostics, synlab genetics s.r.o., Evropska 176/16, Prague 6 16000, Czech Republic; 2 Department I of Internal Medicine, University at Cologne, Kerpener Str., Cologne, Germany; 3 Oncological Therapy Center, Buchforststr., Cologne, Germany; 4 Jena University Hospital, Institute of Human Genetics, Kollegiengasse 10, Jena, Germany

**Keywords:** AML, *MECOM*, Chromosomal microdissection, Next-generation sequencing, Molecular marker

## Abstract

**Background:**

In acute myeloid leukemia (AML), the MDS1 and EVI1 complex locus - *MECOM*, also known as the ecotropic virus integration site 1 - *EVI1*, located in band 3q26, can be rearranged with a variety of partner chromosomes and partner genes. Here we report on a 57-year-old female with AML who presented with the rare translocation t(3;10)(q26;q21) involving the *MECOM* gene. Our aim was to identify the fusion partner on chromosome 10q21 and to characterize the precise nucleotide sequence of the chromosomal breakpoint.

**Methods:**

Cytogenetic and molecular-cytogenetic techniques, chromosome microdissection, next generation sequencing, long-range PCR and direct Sanger sequencing were used to map the chromosomal translocation.

**Results:**

Using a combination of cytogenetic and molecular approaches, we mapped the t(3;10)(q26;q21) to the single nucleotide level, revealing a fusion of the *MECOM* gene (3q26.2) and *C10orf107* (10q21.2).

**Conclusions:**

The approach described here opens up new possibilities in characterizing acquired as well as congenital chromosomal aberrations. In addition, DNA sequences of chromosomal breakpoints may be a useful tool for unique molecular minimal residual disease target identification in acute leukemia patients.

## Background

EVI1 is one of several protein isoforms encoded by the *MECOM* locus at human chromosome 3q26 that also yields the MDS1 and MDS1-EVI1 protein isoform [1]. The role of MDS1 and MDS1-EVI1 in malignancy is still unclear, though the EVI1 transcription factor plays an essential role in the proliferation and maintenance of hematopoietic stem cells [2]. Aberrant EVI1 expression occurs in approximately 8% of patients with *de novo* acute myeloid leukemia (AML) [3]. The overexpression of *EVI1* can be achieved not only through rearrangements of band 3q26 but also without the presence of 3q26 abnormalities, therefore indicating that other mechanisms can lead to *EVI1* activation [4-6]. Moreover, a substantial number of patients with 3q26 rearrangements do not express *EVI1*[7]. In approximately 2% of AML cases, inv(3)(q21q26)/t(3;3)(q21;q26) is observed, where it has been suggested that the promoter of the house-keeping *RPN1* gene could be responsible for the activation of *EVI1*[8]. Other *EVI1* rearrangements include, e.g. 7q21 (*CDK6*), 7q34 (*TCRB*), 12p13 (*ETV6*) and 21q22 (*RUNX1*) [6,9]. Even though partner chromosomes and molecular consequences differ between various types of *EVI1* rearrangements, elevated expression predicts poor prognosis for the affected patients [4,10,11].

Here we report the rare case of chromosomal translocation t(3;10)(q26;q21) involving *MECOM*. Using modern cytogenetic and molecular biological techniques we were able to characterize the nucleotide sequence of this breakpoint and thus identify the fusion partner on chromosome 10.

## Case presentation

A 57-year old female was diagnosed with AML (FAB M2) after a blood cell count and bone marrow examination was initiated in June 2013. Hematologic parameters were as follows: hemoglobin 6,2 g/dl, platelets 44 × 10^9^/l, and white blood cells (WBC) 3,34 × 10^9^/l with 7,8% neutrophils, 62,9% lymphocytes and 28,7% monocytes, 0% eosinophils and 0,3% basophils. A bone marrow aspirate revealed slightly hypercellular marrow with normocellular particles. Megakaryocytes were found in reduced density. There was significant hiatus leucaemicus with evidence of medium-sized blasts with poor basophilic cytoplasm and distinct granulation. Flow cytometry performed on the bone marrow revealed 31% myeloid-appearing blasts with expression of CD34 and CD117, and confirmed the diagnosis of AML.

Conventional cytogenetic analysis of a 24-h culture, performed on bone marrow cells by standard techniques and evaluated by G-banding, revealed a balanced t(3;10)(q26;q21) in 20/22 metaphases. Involvement of the *MECOM* gene was confirmed by FISH with the use of a commercially available probe set.

## Results

**Figure 1:**
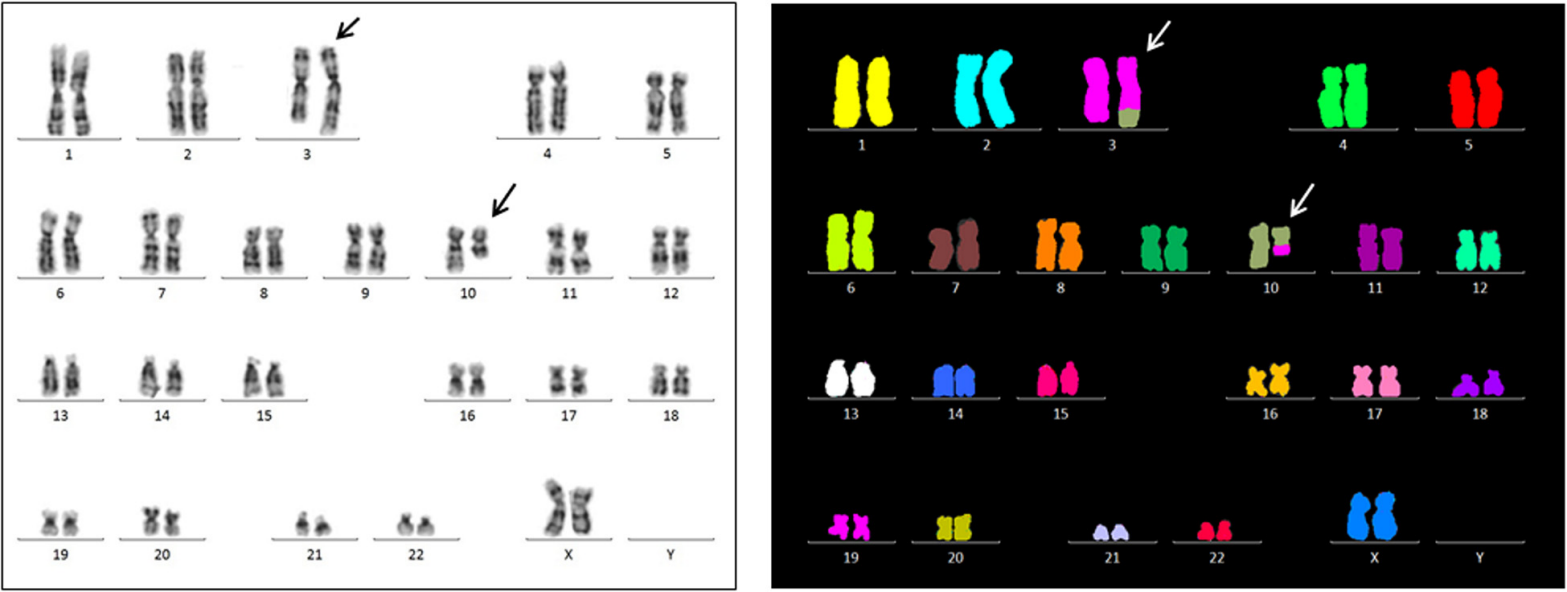
**Karyotype analyses.** G-banding (left part) and multicolor FISH (mFISH) (right part) analyses showed aberrant karyotype 46,XX,t(3;10)(q26;q21). The arrows indicate the derivative chromosomes.

**Figure 2:**
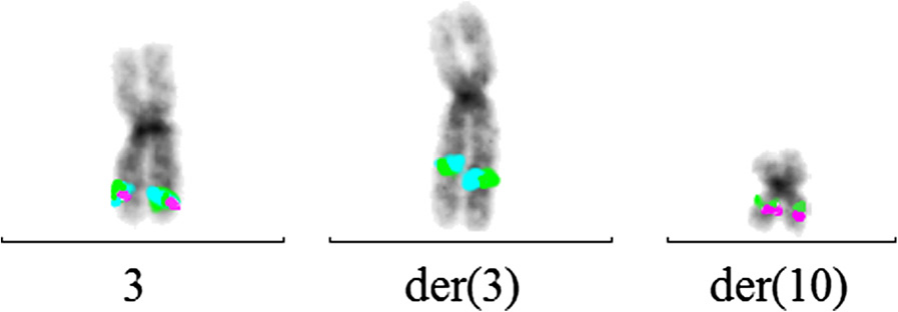
**FISH analysis.** Metaphase-FISH analysis using EVI1 break-apart probe shows normal fusion signal on chromosome 3 (green, purple, blue) and split-signal on der(3) (green, blue) and der(10) (green, purple) indicating rearrangements of 3q26 region.

Cytogenetic and molecular-cytogenetic analyses of bone marrow cultures revealed an aberrant karyotype 46,XX,t(3;10)(q26;q21) – Figure 1. A commercial EVI1 break-apart probe yielded a split signal in all dividing and 80% of the interphase bone marrow cells, demonstrating the rearrangement of the 3q26 chromosomal region (Figure 2).

**Figure 3:**
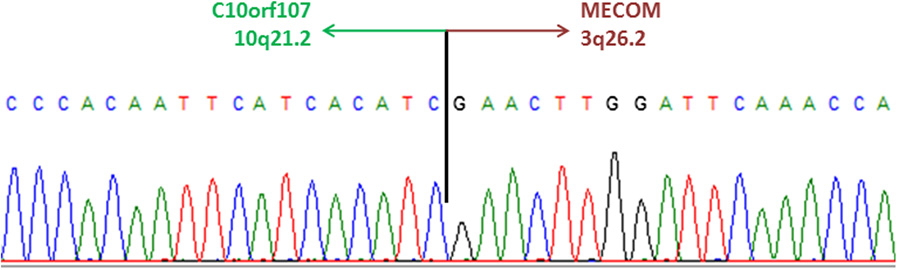
**Derivative chromosome 10 breakpoint sequence.** The electropherogram shows the result of direct sequencing of long-range PCR product which revealed fusion of *MECOM* gene on chromosome 3q26.2 and *C10orf107* on chromosome 10q21.2.

Ten derivative chromosome 10 breakpoint regions were dissected, amplified and sequenced. In total, 81 753 reads were obtained and aligned to reference sequences of chromosomes 3 and 10 (NCBI build 37.3). Long-range PCR primer design resulted in a product that was then subjected to Sanger sequencing. The nucleotide sequence of the der(10) breakpoint (Figure 3) revealed a fusion of the *MECOM* gene on 3q26 to *C10orf107* on 10q21.

Additionally, the bone marrow sample was subjected to reverse transcription real-time PCR analysis to determine the expression levels of cEVI1 (i.e., the sum of all *EVI1* mRNA variants) relative to those of the internal reference gene *ABL*. We found that *EVI1* expression was 26-fold higher when compared with healthy control (data not shown).

## Discussion

In the present report we describe a rare case of acute myeloid leukemia with a t(3;10)(q26;q21) translocation involving *MECOM*. To our knowledge [12], only one case with this translocation has been reported [9], but the fusion partner on chromosome 10 was not characterized. Using a novel technical approach we were able to identify the fusion partner and precise nucleotide sequence of the breakpoint, which may serve as a patient-specific molecular target for subsequent real-time PCR-based minimal residual disease (MRD) monitoring. We further demonstrated by real-time quantitative reverse transcription PCR that the t(3;10)(q26;q21) results in *EVI1* over-expression.

Deregulated expression of *EVI1* and other genes (e.g. *BAALC*, *WT1*) involved in cell proliferation, survival and differentiation have been used as alternative MRD targets [13-16]. However, the sensitivity of expression assays is dependent on the level of initial expression; therefore, these assays are suitable only in AML cases with a high initial expression level of a specific target normalized to an endogenous control gene at diagnosis. Even in those cases, the sensitivity is usually not sufficient for subsequent MRD monitoring. Therefore, in patients presenting with a fusion transcript and/or gene mutation, a specific PCR assay is preferred. These PCR-based methods are currently the most sensitive techniques for MRD follow-up, reaching sensitivities of 10^-4^ – 10^-5^[17,18].

Real-time PCR-based MRD assays allow the highly accurate quantification of residual leukemic cells and evaluations of treatment outcome in AML patients. The merit of MRD monitoring during patient‘s treatment and prognostic relevance has been confirmed by various studies [17,19,20]. Common targets for MRD detection include fusion transcripts (e.g. *RUNX1-RUNX1T1, PML-RARα, DEK-NUP214*, *CBFβ-MYH11*) [21] and mutations of clinically relevant genes (e.g. *NPM1, CEBPα, FLT3*, *c-KIT*) [17-22]. Unfortunately, approximately half of AML patients lack a molecular target suitable for MRD monitoring [23]. Therefore, introducing novel approaches for the identification of unique clone-specific markers is highly desirable. The procedure described here is based on characterizing nucleotide sequences of unique chromosomal breakpoints, allowing the design of a specific real-time PCR assay for MRD assessment. In this way, AML patients could benefit from accurate and sensitive MRD monitoring, even in the absence of other well-introduced molecular marker [24].

Mapping chromosome breakpoints is a conventional method for identifying specific genes in leukemic patients, as well as patients with solid tumors and individuals with balanced translocations [25-27]. A fundamental requirement is the ability to karyotype and precisely identify derivative chromosomes using classic karyotyping or molecular cytogenetic tools such as mFISH and mBAND analyses. Hybridization with even higher resolution, such as BAC-FISH (Bacterial Artificial Chromosome FISH) can help to narrow-down the chromosomal breakpoints further, though it is still not subtle enough to allow subsequent molecular methods to be used and to identify nucleotide sequence. There have been a number of methods proposed to address this issue, with varying strengths and weaknesses. Array-CGH has improved in resolution, allowing deletions, amplifications, and non-balanced translocations to be more precisely characterized, but array-CGH in principle cannot detect targets arising from balanced chromosomal translocations [28].

## Conclusion

The combination of cytogenetic and molecular methods described here enabled us to proceed from the chromosomal level (cytogenetically identified abnormality) to the molecular level (unique DNA sequence) in a case of the novel t(3;10)(q26;q21) translocation. Using this procedure, acquired as well as congenital chromosomal aberrations can be characterized. In contrast to other mapping methods (e.g. BAC-FISH, array CGH) our technique allows the rapid mapping of chromosomal breakpoints down to the DNA sequence level and immediate elucidation of possible genes involved. This can be invaluable for studying such aberrations in a wide variety of fields, including the evolution of diseases or the genetic basis of inherited syndromes.

## Methods

### Cytogenetic and molecular cytogenetic analyses

The heparinized bone marrow sample was cultivated for 24 h in RPMI 1640 media supplemented with 10% fetal calf serum, penicillin/streptomycin and L-glutamine (PAA Laboratories, Austria) at 37°C/5% CO_2_. Karyotype was investigated by G-banding and multiplex fluorescence *in situ* hybridization (mFISH) with the 24XCyte probe kit (MetaSystems, Germany). ISCN 2013 nomenclature was used to describe chromosome abnormalities [29]. Interphase fluorescence *in situ* hybridization (FISH) analysis was performed using a commercially available EVI1 break-apart probe (MetaSystems, Germany).

### DNA/RNA isolation, reverse transcription

DNA and RNA were isolated from the mononuclear fraction of bone marrow samples at diagnosis. DNA was isolated using the MagNA Pure automatic isolator (Roche, Germany) according to the manufacturer’s instructions. RNA was extracted by TRI Reagent (Molecular Research Center, USA) according to the manufacturer’s recommendations. Reverse transcription was performed using the Verso cDNA Synthesis Kit (Thermo Scientific, USA) according to the manufacturer’s instructions.

### Real-time quantitative reverse transcriptase PCR

Primers and probes to amplify and quantify *EVI1*-expression were forward: 5′ ACCCACTCCTTTCTTTATGGACC 3′, reverse: 5′ TGATCAGGCAGTTGGAATTGTG 3′, probe: FAM - 5′ TGAGGCCTTCTCCAGGATTCTTGTTTCAC 3′ - BHQ1. Expression was normalized against the expression of the control gene ABL. Primers and probe to quantify ABL gene were as follows: forward: 5′ TCCTCCAGCTGTTATCTGGAAGA 3′, reverse: 5′ TGGGTCCAGCGAGAAGGTT 3′, probe: FAM-5′ CCAGTAGCATCTGACTTTGAGCCTCAGGG 3′ - BHQ1. PCR conditions started with a denaturation at 95°C for 8 minutes, followed by 45 cycles of denaturation at 95°C for 20 s, annealing at 57°C for 30 s and elongation at 72°C for 30 s.

### Chromosomal breakpoint identification

The cell suspension and DNA sample were treated and analyzed as previously described [24]. Briefly, regions around the breakpoints of derivative chromosomes were dissected by glass microneedles manipulated by micromanipulator using an inverted microscope (Axiovert 10, Zeiss, Germany). The microdissected fragments were directly subjected to amplification by degenerate oligonucleotide-primed (DOP) PCR and then sequenced on the GS Junior platform (Roche, Germany) for next generation sequencing. Obtained reads were aligned to reference sequences of chromosomes 3 and 10, using in-house developed software. The last mapped reads from both chromosomes were used as docking sites for primers for long-range PCR to amplify the putative breakpoint. Primers for long-range PCR were designed in Vector NTI Advance (v. 11.5, Invitrogen, USA). PCR amplification was done using the Expand Long Range dNTPack kit (Roche, Germany). The long-range PCR product was directly sequenced using Sanger sequencing to reveal the precise nucleotide sequence of the breakpoint.

## Consent

Written informed consent was obtained from the patient for publication of this Case Report. A copy of the written consent is available for review by the Editor-in-Chief of this journal.

## Competing interests

The authors declare that they have no competing interests.

## Authors’ contributions

TJ, RP, LZ, DWH, LZ participated in the design of the study and carried out molecular cytogenetic and molecular genetic studies; RP designed the computer software and performed the biostatistical analysis; JS carried out the next-generation sequencing; IP, K-AK, AR performed flow cytometry analysis, collected and provided the clinical data; OAKM, NK performed chromosomal microdissection; SP designed and coordinated the study. All authors read and approved the final manuscript.
